# Transport of Volatiles in Agglutinates from Lunar Regolith of Chang’e-5 Mission

**DOI:** 10.34133/research.0638

**Published:** 2025-03-27

**Authors:** Long Li, Guang Zhang, Hui Zhang, Yuan Xiao, Shaofan Zhao, Jian Song, Wei Yao, Weihua Wang, Zhigang Zou, Mengfei Yang

**Affiliations:** ^1^ Qian Xuesen Laboratory of Space Technology, China Academy of Space Technology, Beijing 100094, China.; ^2^ Department of Aeronautics and Astronautics, Fudan University, Shanghai 200433, China.; ^3^ Beijing National Laboratory for Condensed Matter Physics and Institute of Physics, Chinese Academy of Sciences, Beijing 100190, China.; ^4^ Eco-Materials and Renewable Energy Research Center (ERERC), Jiangsu Key Laboratory for Nano Technology, National Laboratory of Solid-State Microstructures, School of Physics, Nanjing University, Nanjing 210093, China.; ^5^ China Academy of Space Technology, Beijing 100094, China.

## Abstract

Agglutinate particles, an important component resulting from micrometeoroids impacts, account for about 13.4% to 84.7% of the volume of lunar regolith depending on its maturity. They are crucial in the soil’s evolution and the migration of volatile substances. Here, we examined a representative agglutinate particle from Chang′e-5 samples and modeled how volatiles move through its porous framework. Our analysis revealed that the agglutinate’s surface features a patchy distribution of smooth, open pores, as shown by both surface and 3-dimensional structural assessments. By integrating elemental distribution data, we propose that the formation of these smooth, open pores is primarily due to the flow of gaseous volatiles, byproducts of intricate physiochemical reactions occurring in the lunar surface layer during impacts by micrometeoroids. Numerical models of volatile transport in the porous agglutinate have been developed for different flow regimes. These models demonstrate that under the intense conditions of impacts, the transport of volatiles occurs at a remarkably high velocity. Consequently, it is improbable that water would accumulate within the porous structure of lunar soil agglutinates. Nevertheless, understanding this process is valuable for gaining a deeper understanding of the lunar regolith’s development and for potential future endeavors in extracting water from the lunar surface.

## Introduction

To date, over a hundred exploration missions have been conducted to explore the Moon. These endeavors have amassed a wealth of data and have successfully retrieved approximately 384 kg of lunar samples, including regolith, rocks, and dust, and have facilitated the lunar journeys of 12 astronauts. Since the 1970s, research on these samples has established a fundamental scientific comprehension of the Moon’s characteristics [1]. In the 2020s, several nations and organizations have launched ambitious programs around lunar exploration including NASA’s Artemis Plan [2,3], European Space Agency’s “Moon Village” concept [4], International Lunar Research Station (ILRS) program by China National Space Administration (CNSA) [5,6], and Luna-25 [7] and Chandrayaan-3 [8] by Roscosmos and ISRO both for Moon’s south pole landing, and also the exploration plans declared by Japan, South Korea, United Arab Emirates, Israel, and some companies [9–13]. As a major component of lunar soil, the agglutinate particles have been consistently identified in the lunar regolith of all lunar missions. The proportion of agglutinates within lunar soil varies based on the soil’s maturity and particle size, with the potential to constitute up to 40% to 80% of the total soil volume [14–16]. The properties of agglutinate particles are crucial for in situ resource utilization (ISRU) techniques. Specifically, the volume ratio and mineral composition of these particles can impact the spectral absorption and data interpretation from remote sensing, thereby influencing the precision and accuracy of mineral resource detection [17]. Furthermore, mineral paragenesis performances within the agglutinate particles could determine the extraction and beneficiation methods of typical lunar minerals, such as ilmenites, basalts, and metallic particles. Additionally, the agglutinate particles within lunar regolith will also determine the techniques for in situ additive manufacturing and construction with lunar soils [18], as well as the extraction of water and oxygen from lunar soils [19–21], and in situ plant cultivation within it [22]. In summary, understanding the formation processes, morphologies, and mineral composition of agglutinate particles is vital for both lunar scientific research and the implementation of ISRU technologies on the Moon surface.

With the returned samples from the lunar surface by the Apollo program and Luna program, the morphologies, mineral composition, and abundance of agglutinate particles within different lunar soils have been widely reported [23–28]. The mainstream view is that the majority of the agglutinate particles are produced due to the impacts of micrometeorites [27,29]. Detailed investigations have also been conducted on nanophase iron particles within the agglutinate particles. The abundance of nanophase iron particles has been used as a parameter to characterize the maturity of lunar soils [24]. In specific, the higher the maturity of lunar soil, the higher the content of elemental iron in lunar soils. In addition, Li and Milliken [30] found that agglutinate particles might be the main reservoirs of water, with water abundance increasing with the lunar soil maturity. These findings are consistent with the conjectures of water originating from solar wind implantation during agglutinate formation [30–32], which also support the conclusion that solar wind is the dominant source of lunar surface water [30,32]. Daly and Schultz [33] and Honniball et al. [34] further reported that some water molecules may be trapped within the glassy particles following a micrometeorite impaction. Notably, agglutinates usually contain pure iron (Fe) metal nanoparticles and gases implanted by the solar wind, predominantly hydrogen (H) and helium (He), setting them apart from other regolith components [35,36]. Based on previous studies, the most popular model offers some insights into agglutinate formation, which occurs through complex processes following the impact of micrometeoroids on regolith abundant in elements from solar wind. The forceful impacts of the micrometeorites on the lunar regolith generate localized high temperatures and pressures, which melt the mineral fragments, glass grains, and debris of the micrometeorites to form the agglutination particles. Subsequently, water molecules were produced via reactions between solar wind implanted hydrogen and intrinsic oxygen during these impact events [30]. Simultaneously, volatile products, probably gaseous water, resulting from complex chemical processes deplete in the agglutinates and escape into the vacuum of space [37,38]. This explains the dry and barren nature of the lunar regolith, and why investigating the transport of volatiles is crucial to understanding the evolution of lunar regolith. During the Lunar Prospector mission, Kleinhenz et al. [39] conducted experiments on the migration of H₂O in simulated lunar regolith and found that various factors, including exposure temperature, exposure time, and water content, affect the migration and escape rates of H₂O. However, the specific theoretical processes were not elaborated in their work. In Reiss’s research, Knudsen diffusion is considered the dominant process for the thermal escape of volatiles in lunar regolith due to the high vacuum environment of the Moon [40,41]. Teodoro et al. [42] further found that the porous structure of lunar regolith significantly affects the transport characteristics of H₂O within it. In addition, Schieber et al. [43], based on experimental research and previous theoretical findings, conducted a more systematic study on the gas migration process in simulated lunar regolith. They found that the gas migration pattern shifts from continuum flow to free molecular diffusion with increasing Knudsen numbers, and that the porosity and tortuosity of lunar regolith significantly affect the gas transport rate [43,44]. Nevertheless, providing an accurate description of gas volatiles flow within the lunar regolith is of great significance yet very difficult. Theoretically, the description of the full-cycle volatile flow under low-pressure conditions requires a combination of classical continuum flow and molecular flow dynamics. The flow field is often tightly coupled with complex reaction processes and extreme physical conditions in the scenarios of ISRU and lunar regolith evolution [40,43–45]. Technically, the heterogeneous agglutinates pose great challenges for the numerical modeling of the detailed flow field, which strongly affects the macroscopic transport behaviors [46,47]. Thus, an in-depth study of volatile transport within lunar agglutinates, especially the development of an accurate geometric model for the lunar agglutinate particles and numerical model for the volatile transport, would significantly advance the pace of lunar exploration.

On 2020 December 17, the Chang′e-5 (CE5) mission successfully returned approximately 1.7 kg of lunar regolith from the Northern Oceanus Procellarum basin (51.916°W and 43.058°N, a relatively young region compared with those of Luna and Apollo) [48,49]. Within the superficial lunar sample (no. CE5C0400), a significant quantity of agglutinates was observed. The agglutinates, characterized by individual porous particles and extremely irregular shapes, constitute an estimated 45% of the volume of the present lunar soil sample. This study characterizes a representative agglutinate particle from the CE5 superficial samples. Optical microscopy and scanning electron microscopy (SEM) analyses demonstrate that the agglutinate consists of different morphological materials, which make the overall irregular structure. Detailed information from the SEM micrographs indicates very different morphologies between the surface weathered layer and the sidewall of the holes. The agglutinate sample is further analyzed using x-ray computed tomography (X-CT), enabling the creation of a digital geometric model to numerically investigate the transport of volatiles. Finally, computational models for slip flow, transition flow, and Knudsen diffusion are developed to quantify the volatile transport under different pressures and temperatures.

## Results and Discussion

### Morphology and chemical composition of the agglutinate

**Fig. 1.**
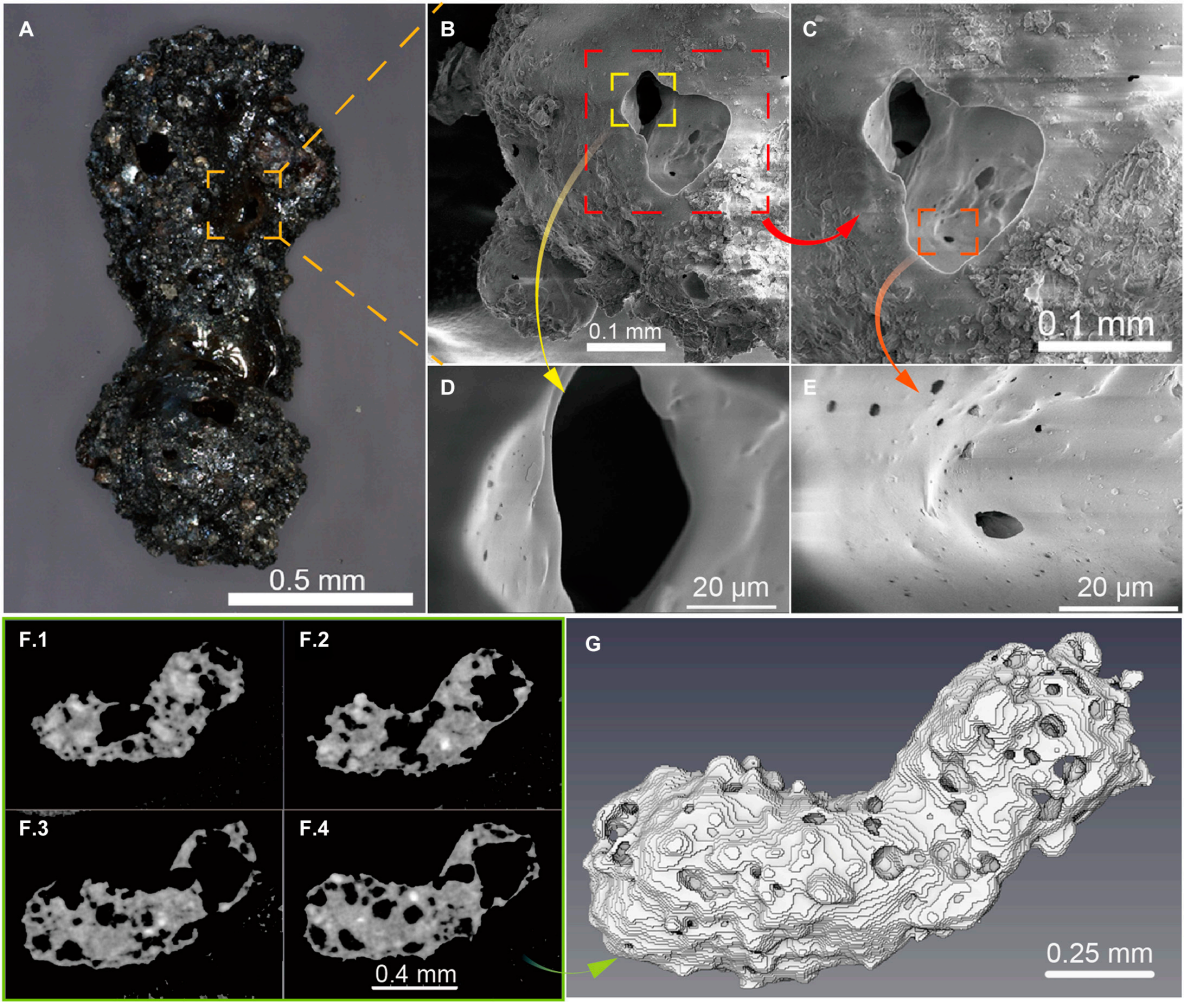
Lunar agglutinate morphology characterization. (A) Optical micrograph of the agglutinate particle. (B) SEM micrograph of the porous agglutinate. (C) Partial enlargement SEM micrograph of the porous agglutinate. (D) SEM micrograph of an opening hole within the agglutinate. (E) SEM micrograph of sidewalls of the opening hole. (F.1 to F.4) Sliced CT images of the agglutinate particle. (G) Reconstructed 3D agglutinate particle.

Microscopic analysis demonstrates that the agglutinate particle exhibits a bi-lobed shape, formed by the adhesion of 2 coarse, dark, porous particles, as illustrated in Fig. 1A. The junction part suggested a smooth, glass-like substance. The agglutinate particle had a length of approximately 1.2 cm and a diameter of 0.4 to 0.7 cm [50]. The optical micrograph with a large depth of field provided a near-3-dimensional (3D) view of the particle’s morphology. A comprehensive 3D reconstruction of the particle’s structure was achieved using X-CT. The sliced CT images of the agglutinate, differentiated by color to represent various minerals (as seen in Fig. 1F.1 to F.4), facilitated the identification of the diverse components within the agglutinate. Agglutinate particles were a common feature across the Apollo and Luna samples. While these particles from different sites share similar traits, the quantity of agglutinates in the CE5 samples varies slightly when compared to other lunar samples. Typically, the individual size of agglutinate particles within the Apollo and Luna samples is under 1 mm [29]. These differences are primarily attributed to the distinct sampling locations and the varying maturity levels of the lunar soil. Reports indicated that agglutinates were formed by random adhesion of small rocks, mineral fragments, and metal particles, creating intricate 3D structures [51–53]. The predominantly black section of the agglutinate indicated that numerous components were entirely enclosed within a dark, glass-like matrix. The exterior surface featured bright and yellow patches of mineral fragments. The microstructures of the agglutinate indicated that the particle originated from the melting soil grains. The fusion and melting of the underlying lunar soil particles were believed to be the result of the intense heat produced by micrometeorite impacts on the lunar surface [14,54–56]. A portion of the melt was ejected as spray during the initial formation of the crater. The remaining impact melt seeped into the gaps between adjacent soil particles, forming a glassy matrix as it cooled and solidified. Throughout this process, volatile elements and water vapor produced by reduction reactions were emitted, leading to the flow of gaseous volatiles through the molten material and the creation of an internal porous structure.

The SEM images of the agglutinate particle are shown in Fig. 1B to E. These pictures display the detailed features of the particles at various magnifications. A globule, displayed in the bottom left corner of Fig. 1B, was believed to have been formed from the bubbling action within molten glass [54,57]. Figure 1B and C reveals numerous minuscule mineral fragments adhering to the particle’s outer surface [50]. In contrast, other fragments on the particle’s exterior surface have been fused closely by the flow of molten glass. Additionally, an open pore was observed within the particle, with a maximum diameter of approximately 0.1 mm, characterized by a smooth and gently sloping side wall. These features indicated that the pore was potentially created by internal gas release, coinciding with the particle’s formation as a result of micrometeorite impacts. The SEM images depicting the interior of the pore and its sidewall are shown in Fig. 1D and E. These pictures further show that the sidewall was smooth with a gentle gradient and feature macropores scattered along its surface. The formation of these macropores was likely also a result of gas volatilization during the agglutinate particle’s formation. SEM and CT images reveal a multitude of intricately connected cavities within the agglutinate, signifying intense gas release during the formation of the particles following micrometeorite impacts. While the specific gases causing pore formation remain undetermined, the prevailing theory among researchers is that they comprise a blend of solar wind-implanted gases, such as hydrogen (H) and helium (He) [58–60].

The characterization of x-ray μ-CT provides a more accurate visualization of the interior structure of the agglutinate particle, revealing the complex interconnections of its pores. Figure 1F.1 to F.4 depicts varied pores within the glassy matrix, observed from multiple perspectives and orientations. The CT analysis revealed that the sample particle has an average pore diameter of about 78 μm and a porosity of approximately 0.71. This porosity is significantly larger than that observed in the porous particle from the Apollo 11 and 14 mission [61], yet it is close to the measurements taken from the upper regolith from the Apollo 11 mission [62]. Figure 1G shows the 3D model of the agglutinate, reconstructed from the X-CT images. The 3D structure shows that the agglutinate particle contained both interior enclosed voids and open pores. The open pores exhibit ellipsoidal or spherical shapes with smooth edges, a feature also observed in SEM images. These characteristics suggest that the voids were generated by the blistering of the components with relatively low boiling points [50], and the agglutinate’s formation was a subsequence of intense volatilization processes.

**Fig. 2.**
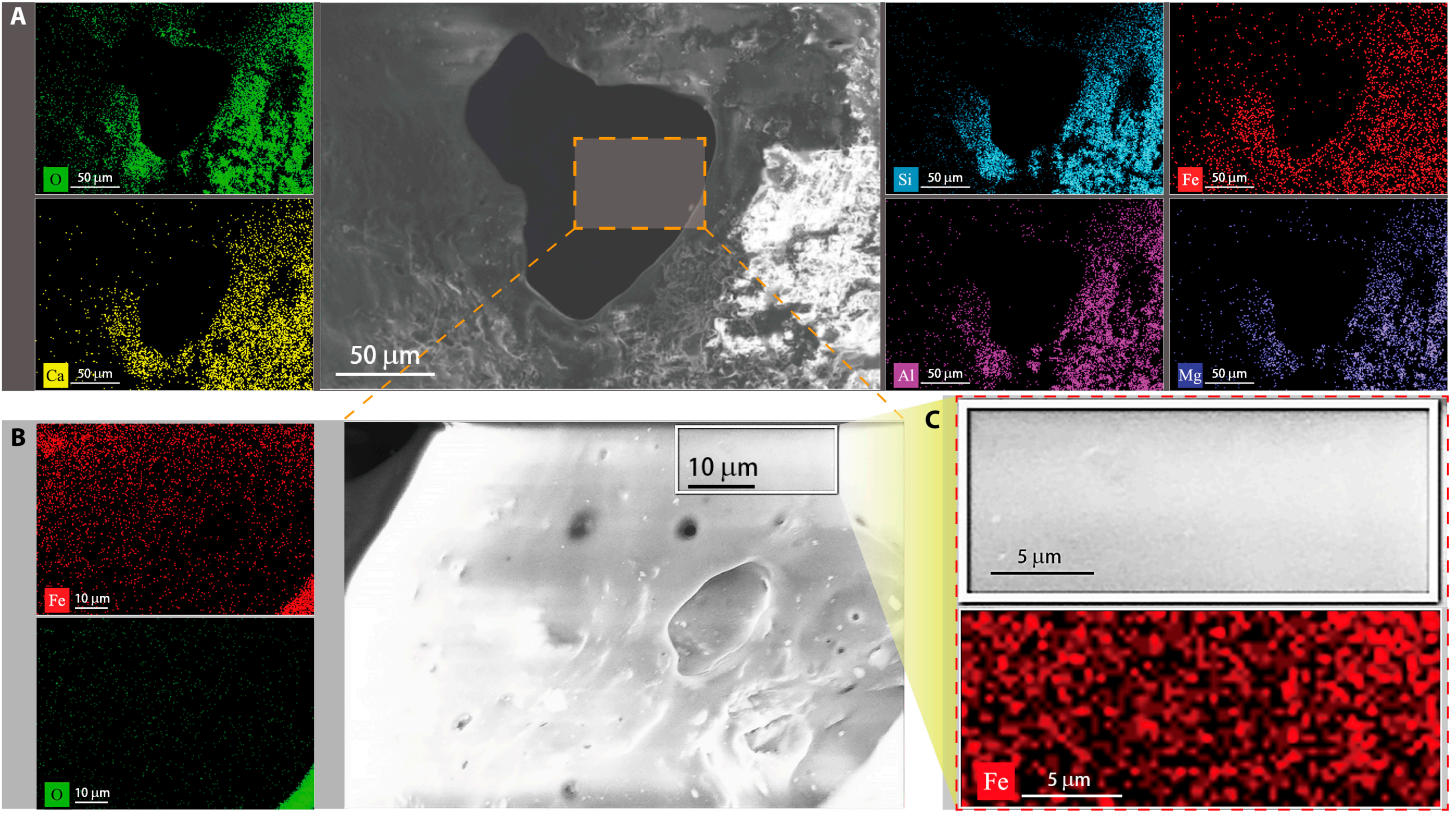
Energy-dispersive spectroscopy (EDS) elemental distributions within the agglutinate. (A) EDS analysis and distribution of main elements (O, Si, Fe, Ca, Al, and Mg) around the surface of agglutinate particle pores. (B) EDS analysis and distribution of main elements (Fe and O) on the smooth sidewall of the zoomed central pore in (A). (C) EDS analysis and iron distribution on the zoomed area in (B).

The x-ray energy-dispersive spectrometry (XEDS) analysis of the particles on the agglutinate’s outer surface indicates a predominance of elements such as oxygen (O), silicon (Si), iron (Fe), calcium (Ca), aluminum (Al), and magnesium (Mg), as illustrated in Fig. 2. Also, trace amounts of titanium (Ti) were also detected. The quantitative elemental characterization is attached in Tables S1 to S3. As previously mentioned, agglutinate particles are typically composed of a variety of rock, minerals, and glass fragments enclosed within impact melt glass [40]. The elemental distribution on the agglutinate’s outer surface shows nonuniform pattern, which is similar to the findings observed in Apollo and Luna mission samples. The types and distribution of elements on the agglutinate’s outer surface mirror those of other CE5 particles, whose dominated elements also include O, Si, Ca, Al, etc. [50]. Figure 2B shows the XEDS scan results of the enlarged view of the open pores at the center of Fig. 2A. The XEDS analysis depicted in Fig. 2B and C reveals a significant difference in content and distribution compared to the adhered mineral fragments in Fig. 2A. Quantitative characterizations demonstrate that the open pores primarily contained elements such as O, Si, Fe, and Al (see Tables S2 and S3). By comparing the elemental distribution on the particle surface and pore wall, it is found that the atomic percent of iron increased by approximately 12 times, while the content of other elements decreased significantly. Therefore, the porous agglutinate particle may harbor a significant quantity of iron-bearing minerals within their internal structure. Furthermore, Fig. 2C depicts the analysis and iron distribution of the zoomed area of Fig. 2B, where particles of pure iron were observed on the sidewall of the open pore on the agglutinate particles. At this stage, the processes and mechanisms that govern the formation of iron particle are still unclear. Regarding the formation of iron in the lunar regolith, there are 3 main hypotheses: the formation of nanophase Fe^0^ from hydrogen reduction of solar wind [63], the large-sized Fe^0^ particles from thermal decomposition induced by the micrometeoroid impact [64], and the mixed Fe^0^ particles from the hydrogen/hydroxyl reduction during micrometeoroid impact [31,65]. In this study, since the agglutinate particles are mainly produced by micrometeorite impacts, and Fe^0^ is concentrated on the sidewalls of the pores, we will focus primarily on the formation of Fe^0^ under the combined effects of micrometeorite impact and hydrogen/hydroxyl reduction. Therefore, pure Fe particles are formed through the following reactions [63]: FeO + H^+^ → Fe^0^+ (-OH)_g_ or FeO + 2H^+^ → Fe^0^ + (H_2_O)_g_. Precipitous and irregular characteristics of the sidewalls of the pores within the agglutinate particle prevented the occurrence of weathering processes. Therefore, it can help preserve the original morphology and distribution characteristics of Fe^0^ particles to the greatest extent. Recently, Li et al. [35] also reported the disproportionation origin of nanophase Fe particles in the CE5 sample, which was attributed to the impacts of micrometeorites. Thus, the preservation of pure iron particles, due to the absence of solar wind irradiation and cosmic radiation, provides valuable insights into the investigation of lunar regolith evolution influenced by micrometeorite impacts. Additionally, it is also observed that bright regions appear at the corners in Fig. 2B, a result of increased atomic layer stacking due to the shorter distance between the hole edge and the x-ray source.

### Numerical investigation of volatile transport through lunar agglutinate

**Fig. 3.**
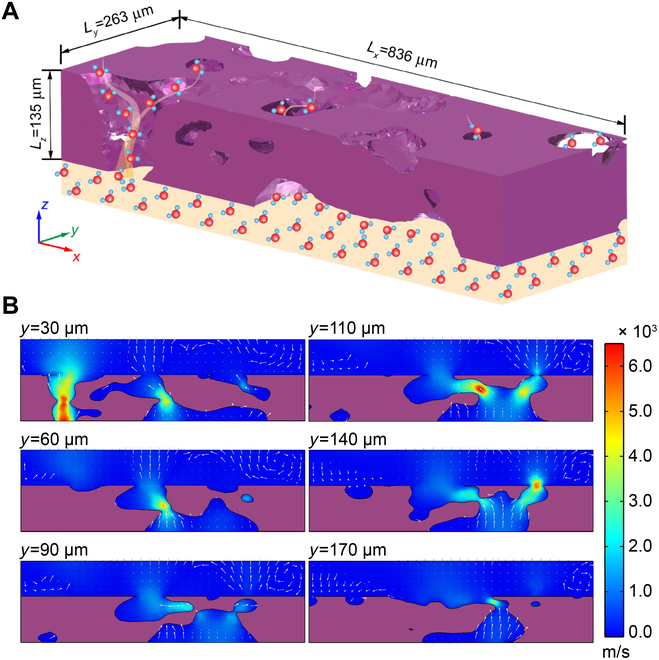
Numerical study of water vapor flow through lunar agglutinate. (A) Results of velocity distribution at different cross-sections with *P*_in_ = 0.95 bar, *T* = 1,500 K. Selection of the cross-sections *y*/*L_y_* = 0.11, 0.23, 0.34, 0.42, 0.53, and 0.65 is based on the interior porous structure. (B) Sketch of water molecules diffusing through the porous Lunar agglutinate. The bottom region where molecules aggregate mimics the originated volatiles from micrometeorite impact.

Direct investigation of volatile transport through the agglutinates is challenging considering the complicated physiochemical interactions between volatiles and solid walls, the extremely irregular morphology, and the flow patterns associated with different Knudsen numbers. For this study, a small rectangular agglutinate domain [region of interest (ROI)] was extracted from the CT reconstructed agglutinate to retain its natural characteristics to the maximum extent and to facilitate the numerical study. Figure 3A depicts the computation model and volatile transport through the complex pore structure. The study considered various flow regimes based on the Knudsen number to determine how local flow distribution is affected by pressure and temperature within the porous agglutinate. Taking the rapid speed and minimal presence of volatiles into account, the potential chemical reactions between volatiles and the solid minerals within the agglutinate particle were neglected (Section S3). Reducing the inlet (bottom surface of the ROI) pressure leads to the transport of volatiles transitioning through slip flow, transition flow, and Knudsen diffusion regime within the agglutinate. Under high inlet pressure conditions ($8,500\leq P_{\mathrm{in}}\leq 95,000$ Pa), the slip flow model was applied to simulate the gas advection flow in the porous structure. The simulation started with an inlet pressure of 8,500 Pa and a reference temperature of 1,300 K. The flow field was initialized with *P*_0_ = 0 and ***u***_0_ = 0. To obtain the detailed flow field through the porous agglutinate, the velocity distribution in the porous structure was examined for *P*_in_ = 0.95 bar, *T* = 1,500 K. The velocity profiles of gaseous volatiles at different cross sections of *y*/*L_y_* = 0.11, 0.23, 0.34, 0.42, 0.53, and 0.65 were illustrated in Fig. 3B, where *y* and *L_y_* are the position and the length of the simulated grain in the *y* direction. It is noted that the velocity field was greatly distorted by the irregularly connected pores, which offer diverse pathways for the volatiles to migrate through the porous agglutinates. Analysis of the velocity field in Fig. 3B revealed that volatiles moved through the porous structure at a mean velocity of approximately 490 m/s. This high velocity is caused by the combined effects of local high-pressure extrusion from impacts and the Moon’s high vacuum suction. In particular, the gaseous flow was severely suppressed and significantly accelerated at the narrow throats, with the maximum velocity potentially reaching 6,500 m/s, further reducing volatile concentrations in the lunar regolith and decreasing the total energy. Once the gas exited the agglutinate and entered the vacuum space, its velocity began to decrease.

**Fig. 4.**
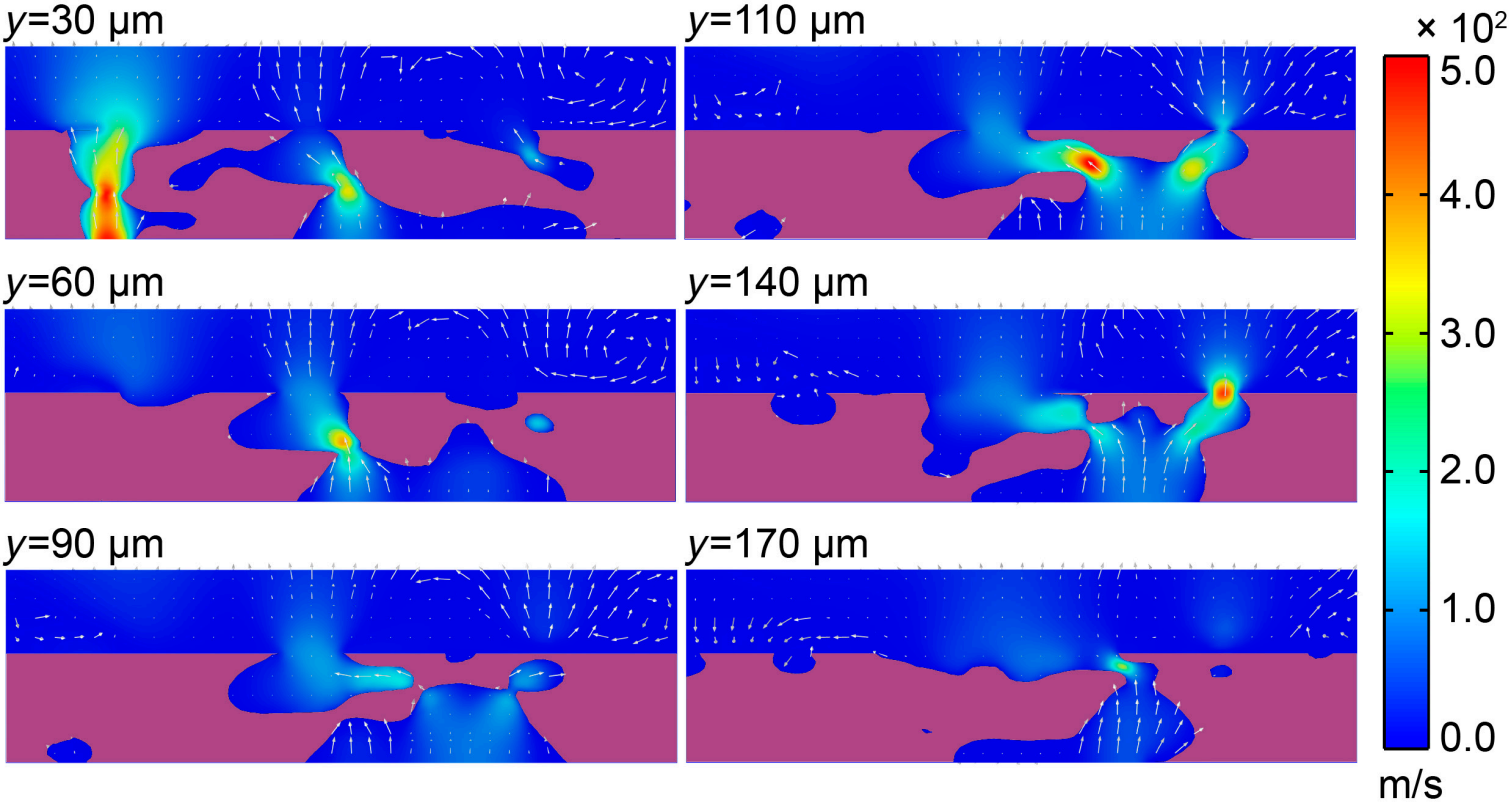
Results of velocity distribution at different cross-sections with *P*_in_ = 8,000 Pa, *T* = 1,500 K. Selection of the cross-sections *y*/*L_y_* = 0.11, 0.23, 0.34, 0.42, 0.53, and 0.65 is based on the interior porous structure.

With the rapid dissipation of gaseous volatiles in the agglutinate particle, further transport of the remaining rarefied gas no longer obeys the continuum hypothesis and the transition flow regime starts to dominate. In the transition flow regime, both the molecular interaction and the molecule–wall interaction play an important role in the volatile transport process. Figure 4 shows the velocity distribution in the porous agglutinate for the transition regime with *P*_in_ = 8,000 Pa and *T* = 1,500 K. The shape of the velocity distribution was similar to that in the slip regime, while the mean speed decreased a lot, with the mean velocity of 43.19 m/s. This is caused by the rising in molecule–wall interaction that makes the blocking effect of irregular pore structure on gas migration more prominent and thus greatly slows the volatile transport as pressure decreased. However, it should be noted that the escape of volatiles from the agglutinate was still very fast. The slip flow and transition flow regimes thus took away a large portion of volatiles in a very short time.

**Fig. 5.**
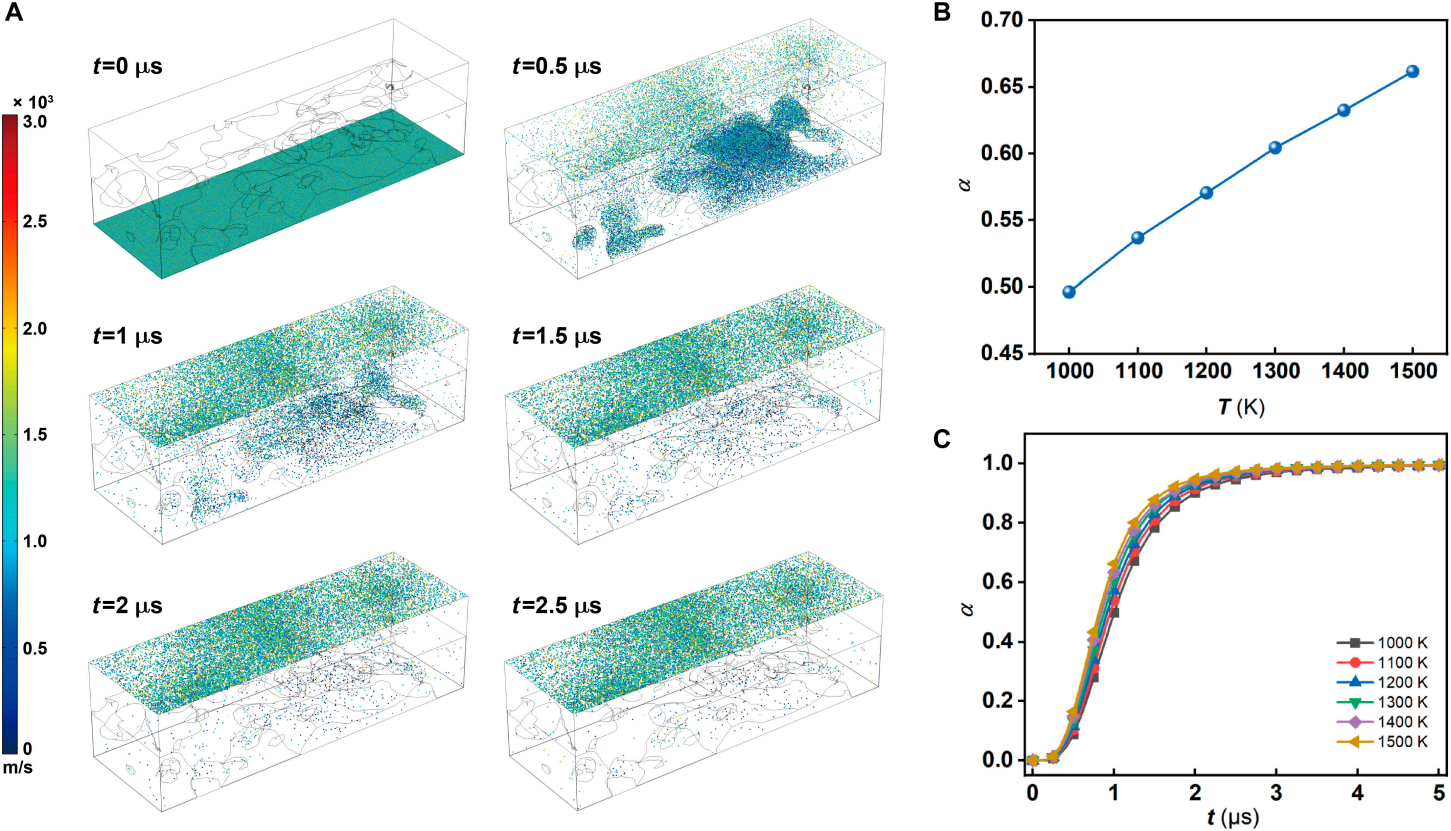
Results of Knudsen flow through lunar agglutinate. (A) Molecule distribution at *t* = 0, 0.5, 1.0, 1.5, 2.0, and 2.5 μs. Color legend represents velocity magnitude. (B) Transmission probability with temperature at *t* = 1 μs. (C) Transmission probability with time for different temperatures.

As the inlet pressure continuously decreases, the molecule–wall interactions become dominant over intermolecular interaction and the Knudsen diffusion regime dominates volatile transport. In the Knudsen diffusion, the gas molecules were fixed at the bottom layer to mimic the volatiles underneath. Once released, volatile molecules rapidly diffused through the porous medium and piled up at the throat regions as illustrated in Fig. 5A. It is noted that the time of gas transport through the porous sample under the Knudsen diffusion regime was on the order of 1 μs, which is much longer than the continuum regime. Gas molecules gathered at the bottom pores and diffused into the sample after initially being released. At *t* = 0.5 μs, a large portion of the gas molecules were still piled at the bottom inlet pores for the system temperature *T* = 1,500 K, and only a small number of gas molecules were transported through the sample. The transmission probability (*α*) measuring the percentage of gas molecules transported through the sample was approximately 0.165 (Fig. 5C). As depicted in Fig. 5A, the gas molecules migrated with a mean velocity much slower than that in the slip and transitional flow regimes. It should be noted that the color legend of velocity in Fig. 5A represents the particle velocity magnitude, which is only the function of temperature. The mean velocity was thus evaluated by calculating the total time for molecules traveling through the agglutinate. Due to the large mean free path of the rarefied volatiles, gas molecules interacted frequently with the solid surfaces instead of self-interactions. Therefore, the molecule diffusion inside the porous medium was greatly decelerated due to the irregular scattering process. At *t* = 1.0 μs, more than a half portion of the gas molecules was transported through the porous sample, while the diffusion process greatly slowed down after *t* = 1.5 μs. Although the residual gas molecules in the porous agglutinate were less and less after 2 μs, it was difficult to expel them completely and some volatiles might be permanently preserved in the agglutinates and glass beads [66]. Combining with the element distribution, the transport behavior of gaseous volatiles in the porous agglutinate could provide more details on the evolution of the barren Moon regolith and offer ideas for the construction of the artificial environment of the future Lunar research station.

To evaluate the statistical pattern of the volatile diffusion via the agglutinate, we computed the gas transmission probability variation under different temperatures. In Fig. 5B, it is found that the gas transmission probability monotonically increased with temperature. As the surrounding temperature increased, the mean kinetic energy of gas molecules also increased according to ${\bar{\varepsilon }}_{k}=\frac{1}{2}m{\bar{v}}^{2}=\frac{3}{2}\mathrm{kT}$ from statistical thermodynamics, which subsequently increased the probability of gas molecules being transported through the porous sample. As indicated in Fig. 5B, the transmission probability increased approximately 33.30% as temperature increased from 1,000 to 1,500 K at *t* = 1 μs. It is noteworthy that the slope of the transmission probability curve decreased as temperature increased. This is mainly caused by the surface mitigation as a result of molecule–surface scattering. Generally, the gas molecules diffuse through the porous sample under both the molecule–wall interaction and self-interaction. As the temperature increased, the extra kinetic energy would bolster the microscopic interactions and more gas molecules tended to get through the porous region. Therefore, the number density of gas molecules in the porous region decreased. The diffusion of gas molecules through the porous sample was greatly mitigated by the irregular pore geometry and rarefied gas density, as illustrated in Fig. 5A and C. The curves of transmission probability variation could be divided into 3 stages by slope. At the initial stage, *α* slowly increased with time and was lower than 0.015. This stage corresponded to the entrance of gas molecules into the porous agglutinate, and the volatiles were greatly confined by the porous structure. Once the gas molecules fully occupied the pore space and diffused to the outlet boundary, they began to escape the porous sample and rushed into the vacuum space. This corresponded to the middle stage and *α* sharply increased with time. In the last stage, *α* gradually increased with time and approached 1, which represents the slow diffusion process due to the rarefied gas density and gas–wall scattering. For T≥1,000K, *α* increased to 0.9 in 2.1 μs. Nevertheless, it was difficult to get rid of all the gas molecules in the porous region.

**Fig. 6.**
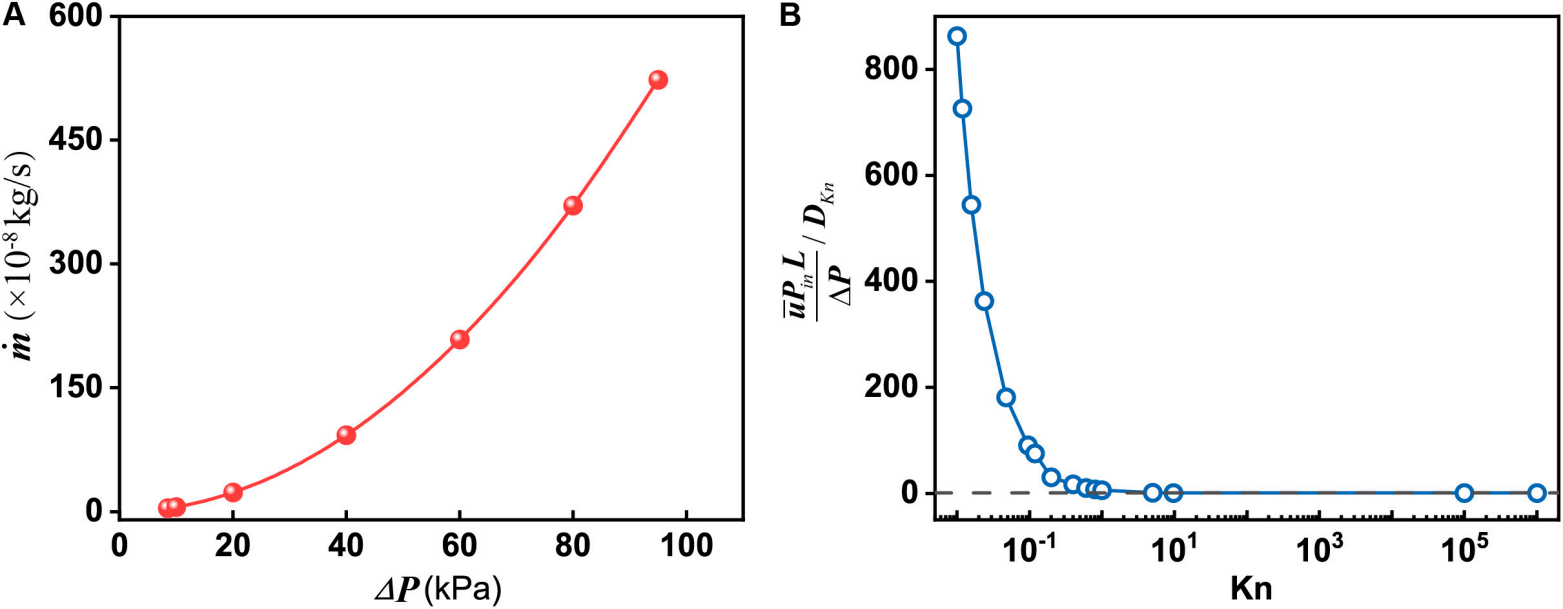
The mass flow rate in different flow regimes. (A) Mass flow rate to pressure difference for slip flow. (B) Normalized mass flow rate to Knudsen number.

The evaluated mass flow rate for different flow regimes is shown in Fig. 6. For slip flow and transitional flow, the mass flow rate can be quantitatively calculated by the continuity equation in Eq. 1.

$$\dot{m}=\bar{u}\rho A_{c}$$ (1)

where $\bar{u}$ is the average superficial velocity at the inlet of the porous medium, *ρ* is the fluid density, and *A*_c_ is the total cross-section area of the flow field [43]. The average superficial velocity can be evaluated by the computed velocity distribution from the Navier–Stokes equation. In the advection flow regime under the continuum theory framework, the produced volatiles escaped through the porous agglutinate on the order of 10^−8^ to 10^−6^ kg/s for different pressure conditions and the flow rate increased nonlinearly with pressure difference (Fig. 6A). The dependence of flow rate on the pressure difference could be qualitatively predicted by the Hagen–Poiseuille equation, and the nonlinearity was caused by the pressure-dependent gas density and slippage condition in the porous structure. To study the overall flow properties under different flow regimes, the normalized mass flow rate was evaluated with Kn as illustrated in Fig. 6B. $\frac{\bar{u}P_{\text{in}}L}{\Delta P}$ represents the mass flow rate over pressure gradient and is normalized to the Knudsen diffusivity $D_{\mathrm{Kn}}=\frac{2}{3}\frac{\phi }{\tau }\sqrt{\frac{2\mathrm{RT}}{\pi M}}{\bar{d}}_{w}$, where ${\bar{d}}_{w}$, ϕ, and τ are the parameters of the porous particle and represent the mean pore diameter, the porosity, and the tortuosity, respectively; *R* is the universal gas constant; and *M* is the molar mass of the volatile [67]. The normalized mass flow rate for the Knudsen diffusion regime was set to 1 for the same temperature. For 0.01 < Kn < 0.1, the normalized $\frac{\bar{u}P_{\text{in}}L}{\Delta P}$ drastically decreased with the decreasing pressure as expected, which is also consistent with the experimental conclusions of Schieber et al. [43]. Since the flow region in this study is limited to the part of a single agglutinate particle (with very low flow resistance), and its structure is significantly different from that in the literature, the normalized flow rate is one order of magnitude higher in comparison. While the normalized $\frac{\bar{u}P_{\text{in}}L}{\Delta P}$ smoothly decreased from approximately 75 to 1 as the Kn increased from 0.12 to 9.67, the transitional flow regime demonstrated a smooth bridge between the steep decrease in slip flow regime and the leveling out in Knudsen diffusion. The flow rate at this stage is in good agreement with the experimental results in the literature [43]. This is because, during the Knudsen diffusion stage, the gas migration rate is mainly determined by the collisions between the fluid molecules and the flow structure, as well as the system temperature. The flow region in this study is derived from the agglutinate particle of real lunar regolith, which is quite similar to the simulated lunar regolith in the literature in terms of porosity and pore size. Of course, the system temperature in this study is much higher than the range in the literature, resulting in an increase in flow rate. Given the limited number of volatiles and high vacuum conditions on the Moon, the gas flow was bound to be quite fast and the upstream pressure decreased very quickly until the continuum theory failed and Knudsen diffusion dominated. High vacuum conditions accelerated the escape of volatiles and made the Moon’s surface even barren unless the volatiles were trapped by enclosed glass beads.

## Conclusion

Agglutinates, serving as pivotal indicators of lunar soil maturity, are instrumental in deciphering the progression of lunar soil and advancing ISRU technologies for forthcoming lunar expeditions. A CE5 sample was chosen for comprehensive analysis, featuring an agglutinate particle of a larger size than those documented in Apollo and Luna missions, to explore its microstructure, mineral content, and capacity for volatile transport. Initial characterization of the agglutinate particle was performed using optical microscopy, SEM, and X-CT. These analyses suggested that the smooth, porous structure likely resulted from the flow of volatiles. The elemental distribution analysis supported the hypothesis that water vapor was a primary volatile component, inferred from the reduction in FeO caused by impacts from micrometeorites. Subsequently, computational models for slip flow, transition flow, and Knudsen diffusion were formulated to elucidate volatile transport across varying pressures and temperatures. The findings indicated rapid volatile escape, rendering the upper regolith layer dry and devoid of moisture under high-vacuum conditions. Within the slip flow regime, the average velocity of volatiles escaping through the porous agglutinate could reach speeds of up to 490 m/s. In the Knudsen diffusion regime, *v*, the diffusion of volatiles was temperature-dependent, with the majority of volatiles traversing the agglutinate within 5 μs. It should be noted that this study did not account for chemical reactions during volatile transport due to their complex influence on the flow dynamics and the porous structure of the agglutinate particles. Practically, water vapor might be adsorbed by nearby surfaces before dispersion, a phenomenon referred to as residence time, which could partially decelerate the escape of volatiles. Nevertheless, the adsorption process could be greatly reduced at elevated temperatures, potentially enhancing reverse reactions between water vapor and mineral surfaces. This will further complicate the problem and is therefore out of the scope of this work. Despite agglutinates being potentially depleted sources of water and other volatiles, they offer a rich source of iron and valuable insights into the study of lunar regolith evolution.

## Materials and Methods

### Sample preparation and procedure

A porous agglutinate particle was selected from the CE5’ surficial lunar samples (CE5C0400) allocated by the CNSA. These samples were sealed and transported in a nitrogen bottle and stored in a nitrogen glovebox to avoid the influence of the earth’s atmospheric environment. All the measurements were carried out in the class 1000 clean laboratory. Before the x-ray μ-CT and the SEM-XEDS scanning characterization, the apparent morphology of the agglutinate particle was characterized by an auto-focusing microscope (ZEISS Smartzoom5), and the optical micrographs obtained with a large depth of field are shown in Fig. 1A.

### X-ray μ-CT

The x-ray μ-CT is widely used in the research of geology, minerals, extraterrestrial soils, and rocks. In this study, a high-resolution X-CT system (Phoenix v|tome|x s240 nano/micro-CT, GE Sensing & Inspection Technologies GmbH) was used for the characterization of the 3D geometry of the agglutinate particle. The scanning recipe of 180 kV and current of 100 μA, for a power of 18 W, were applied for all scanning tests. The particle sample was first transferred from the glove box and sealed in a nitrogen bottle, and then the sample bottle was placed on a customized thin rod tray to ensure a distance of approximately 10 mm away from the x-ray source. The distance between the x-ray source and the detector was 500 mm. A 1-mm-thick copper sheet was placed in front of the source for filtration. This process removes the low-energy radiation, retaining only the high-energy rays to maintain consistent penetrating power, and the pixel pitch for the detector was 200 μm, which resulted in a detection voxel size of 4 μm. For the agglutinate sample, a total of 1,000 images were collected through 360° rotations, using the exposure time of 1,000 ms for each image and averaging with 3 images. Before each group of scans, a bias and gain calibration and correction were completed. The collected images were processed using Phoenix datos|x 2.0 CT software (GE Sensing & Inspection Technologies GmbH) for initial projection reconstruction, and further processing, including segmentation, quantitative data measurements and analysis, and geometry meshing, was completed in VGStudio Max 2.4 software (Volume Graphics GmbH). By superimposing different CT scan slice images, it is possible to achieve a 3D reconstruction of the CT image. For the verification of the accuracy of 3D CT reconstruction images, the length and volume of a reference sample were measured using the 3D reconstruction system and evaluated with standard data, ensuring that the average error of the test results is less than 1%. For the present study, pores with dimensions larger than 5 μm could be accurately identified with the 4-μm voxel size used in the scanning recipe. Finally, the ROI region was carefully extracted from the 3D sample geometry, ensuring as much as possible that the average porosity and overall shape of both are consistent, as illustrated in the Supplementary Materials. The meshing file of the ROI geometry was subsequently generated for further numerical study in COMSOL Multiphysics 6.0 software (COMSOL Inc.).

### Sample sectioning and SEM-EDS scanning

Based on the optical morphology of the agglutinate particle, the smooth areas of the particle surface were selected as the SEM-EDS test and analysis area. The sample was first pasted to a sample table by a conductive adhesive. Then, the sample was put into the vacuum test chamber, and the electronic excitation voltage was increased to about 20 kV. The SEM micrograph of the porous agglutinate particle, the partial enlargement SEM micrograph of the porous agglutinate particle, the SEM micrograph of an opening hole within the agglutinate particle, and the SEM micrograph of the sidewalls of the opening hole were obtained (Fig. 1B to E). At the same time, the chemical composition of these different regions on the agglutinate particle was detected using the EDS installed in the SEM (Fig. 2).

### Computation method

As is reported in previous studies, the volatiles existing in the agglutinates are mainly formed by the reduction of FeO compound due to impacts of micrometeoroids into lunar regolith, which contains abundant H and He after long-time exposure to the solar wind. Porous agglutinates and iron are created, and most of the volatiles escape from the agglutinates after the impacts. Additionally, thermal decomposition reactions during impacts may produce some O₂. The volatile components formed by these gases have a significant impact on the formation of porous cement in lunar soil. In this study, to simplify the analysis, the chemical processes involved in the migration of volatile components are neglected. Instead, we mainly investigate the kinetic laws of gas transfer caused by factors such as pressure and temperature. Furthermore, based on previous studies, it is noted that water vapor might have a higher proportion compared to other components in the volatiles, and its molecular weight is moderate. Therefore, we selected single-component water vapor as the working fluid for simulation studies. To accurately simulate the transport process of volatiles in agglutinates, the flow conditions must be carefully evaluated. The volatile flow through the porous structure can be evaluated by the gas flow within small channels, where the velocity distribution and flow pattern greatly depend on the inlet pressure and environment temperature [40,43,68]. Taking into account the high vacuum environment on the moon and the local high temperature and high pressure resulting from micrometeorite impacts, the volatiles, once produced, will be driven outward into the external vacuum by the large pressure difference, continuously reducing the pressure in the fluid domain until the internal and external pressures are equal, namely, until all volatiles have escaped. Therefore, the flow pattern of volatiles in this process can be assessed by calculating the Knudsen number of the flow domain. Generally speaking, at the initial moment, the high temperature and high pressure of local volatiles will make the Kn number less than 0.1, and the fluid flow can be simulated using the continuum flow model and the Navier–Stokes equations. To simulate the high temperature and high pressure after micrometeorite impacts, the temperatures between 1,300 and 1,500 K and pressures between 8,500 and 95,000 Pa are selected as the initial conditions for volatiles, ensuring that the Knudsen number is within the range of 0.01 to 0.1. Then, the slip flow theory is employed to perform numerical calculations on the transport dynamics of volatiles. As the pressure of the flow domain continuously decreases, transition flow and Knudsen diffusion are chosen accordingly to quantify the flow dynamics under transition flow and free molecular flow regimes [43]. Specifically, the Monte Carlo method is used to statistically analyze the microscopic motion of molecules and their collisions with walls, thereby obtaining the non-equilibrium flow characteristics of rarefied gases. Commercial finite element software, COMSOL Multiphysics 6.0, on Dell Precision T7820 operated with Ubuntu 17.10 was used to conduct the simulations. The computation system consists of a 3D porous agglutinate sample and volatile molecules. The porous agglutinate is taken from the CT model of agglutinates with a volume of 836 × 263 × 135 μm^3^, and the volatile molecules are released from the bottom of the system and diffuse through the micropores into the vacuum zone on top of the sample, as depicted in Fig. 3A. Detailed computation methods can be found in Section S4.

## Data Availability

All data generated and analyzed in this study are included in the article. A complete dataset for this study is also available at Mendeley Data at https://data.mendeley.com/drafts/zh566str8p.

## Supplementary Materials

Supplementary 1 Sections S1 to S4 Figs. S1 to S5 Tables S1 to S6
