# Supplementary material for: Transport of Volatiles in Agglutinates from Lunar Regolith of Chang’e-5 Mission
Source: Research (Wash D C). 2025 Mar 27;8:0638. doi: 10.34133/research.0638 (PMC11948346; doi:10.34133/research.0638)
Supplement: Supplementary 1 — Sections S1 to S4 Figs. S1 to S5 Tables S1 to S6 [file research.0638.f1.docx]

**Supplementary Information**

**Transport of Volatiles in Agglutinates from Lunar Regolith of Chang’E-5 Mission**

Long Li^1,2†^, Guang Zhang^1,†^, Hui Zhang^1^, Yuan Xiao^1^, Shaofan Zhao^1^, Jian Song^1^, Wei Yao^1,*^, Weihua Wang^1,3^, Zhigang Zou^1,4^, Mengfei Yang^5,*^

*^1^Qian Xuesen Laboratory of Space Technology, China Academy of Space Technology, NO. 104 Youyi Road, Haidian District, Beijing 100094, China*

*^2^Department of Aeronautics and Astronautics, Fudan University, NO. 220 Handan Road, Yangpu District, Shanghai 200433, China*

*^3^Beijing National Laboratory for Condensed Matter Physics and Institute of Physics, Chinese Academy of Sciences, Beijing 100190, China*

*^4^Eco-Materials and Renewable Energy Research Center (ERERC), Jiangsu Key Laboratory for Nano Technology, National Laboratory of Solid-State Microstructures, School of Physics, Nanjing University, Nanjing, 210093, China*

*^5^China Academy of Space Technology, NO. 104 Youyi Road, Haidian District, Beijing 100094, China*

1. **Characterization Lunar Regolith Samples**

SEM and XEDS analysis were also carried out for the characterization of the rock particle from the CE5C0400 sample. The top panel of Fig. S1 demonstrates the SEM picture of the rock particle agglutinate particle. The appearance showed a solid particle with a weathered rough surface. Fig S1b reveals that the rock particle was comprised primarily of oxygen (O), silicon (Si), iron (Fe), calcium (Ca), aluminum (Al), and magnesium (Mg) elements. The detailed mineral composition distribution data is listed in Table S4. It is observed that the distribution of elements on the rock particle is similar to the results of the agglutinate particle. The detailed mineral composition distribution data corresponding to Fig. 2a is shown in Table S1. For comparison, we also conducted a statistical analysis of the element distribution in Figures 2b and 2c. As shown in tables S1-3, the element distribution on the pore walls is significantly different from that on the particle surfaces, with a marked increase in the content of elemental Fe.


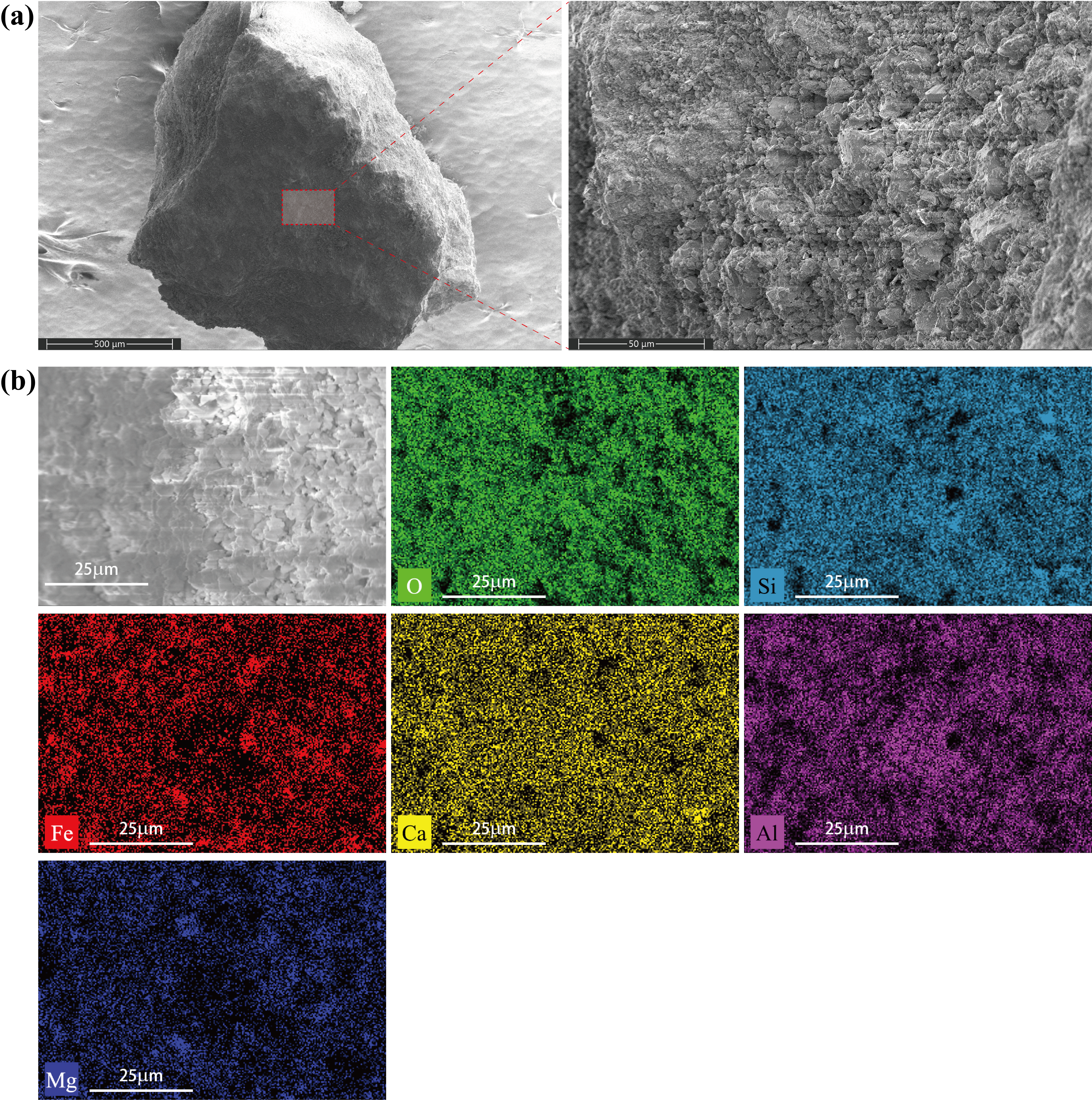


**Fig. S1** SEM (a) and energy dispersive spectroscopy (EDS) elemental distributions (b) of the rock particle from CE5C0400.

**Table S1.** The detailed mineral composition corresponding to the mapping region in **Fig. 2a.**

| **Element** | **wt%** | **Atomic Percent** |
| --- | --- | --- |
| O | 58.40 | 74.71 |
| Mg | 3.21 | 2.70 |
| Al | 5.24 | 3.97 |
| Si | 15.22 | 11.09 |
| Ca | 5.86 | 2.99 |
| Ti | 1.81 | 0.77 |
| Fe | 10.26 | 3.76 |
| Total | 100.00 | 100.00 |

**Table S2.** The detailed mineral composition corresponding to the mapping region in **Fig. 2b.**

| **Element** | **wt%** | **Atomic Percent** |
| --- | --- | --- |
| O | 36.76 | 60.93 |
| Mg | 2.32 | 2.53 |
| Al | 3.22 | 3.16 |
| Si | 10 | 9.44 |
| Ca | 5.60 | 3.71 |
| Ti | 3.13 | 1.73 |
| Fe | 38.97 | 18.50 |
| Total | 100.00 | 100.00 |

**Table S3.** The detailed mineral composition corresponding to the mapping region in **Fig. 2c.**

| **Element** | **wt%** | **Atomic Percent** |
| --- | --- | --- |
| O | 14.47 | 34.30 |
| Mg | 1.58 | 2.46 |
| Al | 1.80 | 2.53 |
| Si | 5.30 | 7.15 |
| Ca | 3.88 | 3.68 |
| Ti | 2.94 | 2.32 |
| Fe | 70.04 | 47.57 |
| Total | 100.00 | 100.00 |

**Table S4.** The detailed mineral composition corresponding to the mapping region in **Fig. S1.**

| **Element** | **wt%** | **Atomic Percent** |
| --- | --- | --- |
| O | 54.66 | 70.97 |
| Mg | 3.01 | 2.57 |
| Al | 8.41 | 6.48 |
| Si | 16.12 | 11.94 |
| Ca | 6.52 | 3.38 |
| Ti | 1.90 | 0.82 |
| Fe | 8.32 | 3.09 |
| Na | 0.46 | 0.41 |
| P | 0.13 | 0.10 |
| S | 0.15 | 0.10 |
| K | 0.17 | 0.10 |
| Mn | 0.13 | 0.05 |
| Total | 100.00 | 100.00 |

In addition, we have also examined different regions of the pore surface within the agglutinate particle shown in Fig. 2b, as illustrated in figure S2. A quantitative analysis of the elemental distribution revealed that the results are similar with Fig. 2c.


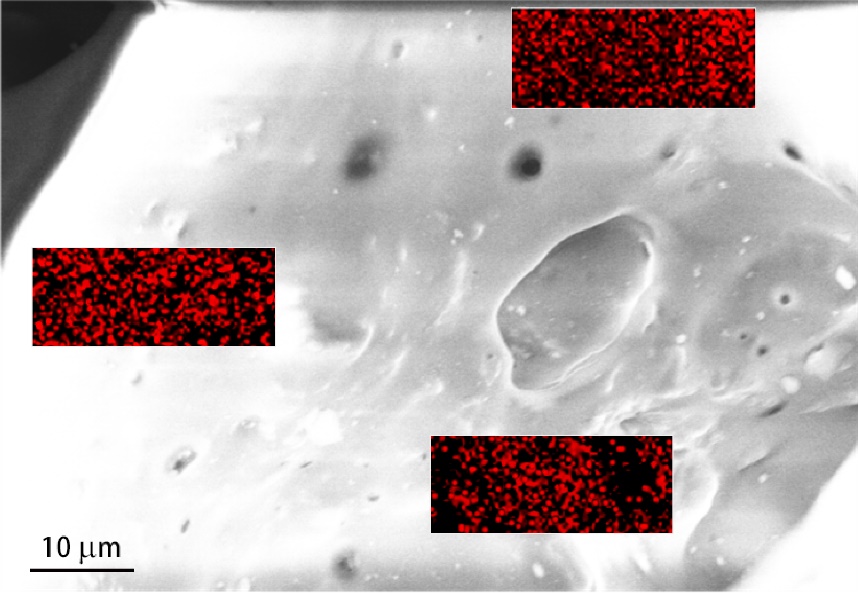


**Fig. S2** Energy dispersive spectroscopy elemental distributions for different regions of the open pore surface within the agglutinate particle from CE5C0400.

**Table S5.** The detailed mineral composition corresponding to the mapping region in **Fig. S2.** Region 1-3 correspond to the upper-right, middle-left, and lower-right insets of Figure S2.

| **Element** | **Atomic Percent** | | | |
| --- | --- | --- | --- | --- |
|  | **Region 1** | **Region 2** | | **Region 3** |
| O | 34.30 | 33.21 | | 38.63 |
| Mg | 2.46 | 2.78 | | 3.12 |
| Al | 2.53 | 2.26 | | 2.88 |
| Si | 7.15 | 6.73 | | 8.03 |
| Ca | 3.68 | 3.54 | | 3.71 |
| Ti | 2.32 | 2.17 | | 2.59 |
| Fe | 47.57 | 49.31 | | 41.04 |
| Total | 100.00 | 100.00 | 100.00 | |

After the characterization of the agglutinate particle by CT, 1000 slice images were superimposed to achieve a 3D reconstruction of the CT image for the porous agglutinate. As for the selection of the Region of Interest (ROI), we directly cropped it from the scanned sample image, ensuring as much as possible that the average porosity and overall shape of both are consistent (flat and elongated). The following figure illustrates the cropping area (in blue) within the overall structure of the sample.


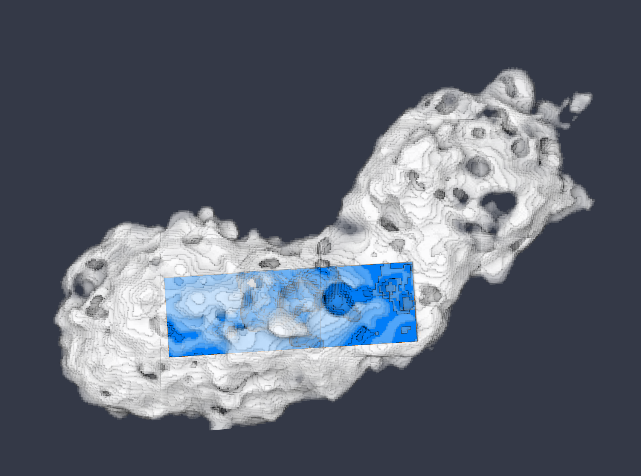

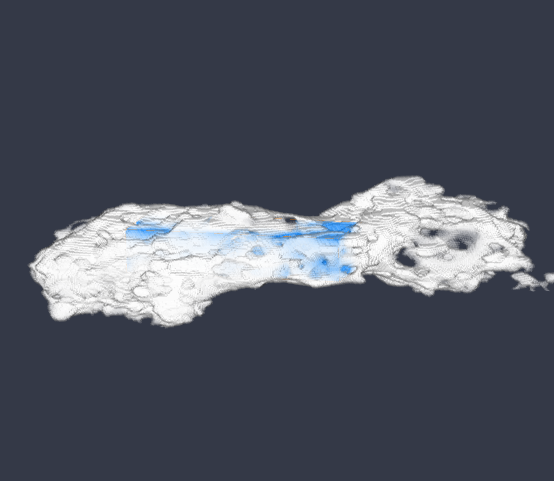


**Fig. S3** Selection of ROI within the three-dimensional reconstruction of the agglutinate particle. (a) top view and (b) side view, with blue box representing the cropped ROI region.

1. **Comparison of Present Agglutinates with Other Missions**

Agglutinate particles were widely found in the Apollo samples and Luna samples. The amount of agglutinate particles in these lunar samples is slightly different from the CE5 samples. The size of individual agglutinate particles in the Apollo samples and Luna samples is typically less than 1mm. However, the detailed elemental distributions at different positions of agglutinate particles were not fully tested and analyzed previously. The volume ratio of agglutinates in these lunar soils was investigated in detail and the influence on the soil maturity was analyzed. The major conclusions could be summarized as follows:

1. The formation of agglutinates in lunar soils (from micrometeorite impact) results in significant fractionation of the major elements;
2. Lunar soil maturity can be evaluated by measuring modal and particle contents (agglutinates);
3. The agglutinate contents show a general decrease with depth, and the 90-150 μm fraction contains a greater percentage of agglutinate than the 150-250 μm fraction.

**Table S6** Comparison of agglutinate fractions in different lunar soil samples.

| **References** | **Mission**  **（Sample ID）** | **Particle size**  **(μm)** | **Agglutinates amount (%)** |
| --- | --- | --- | --- |
| Ref. 1 | Apollo 14 (sample14240,17) | / | 23% |
|  | Apollo 15 (sample15100,9) | / | 48% |
|  | Apollo 17 (sample72501,56) | / | 54% |
|  | Apollo 17 (sample72141,16) | / | 63% |
| Ref. 2 | Apollo 16 (sample61241, 26) | / | 57.1% |
|  | Apollo 16 (sample61501, 15) | / | 55.7% |
|  | Apollo 16 (sample63321, 16） | / | 35.5% |
|  | Apollo 16 (sample63341, 19-21) | / | 33.6% |
|  | Apollo 16 (sample64421, 18) | / | 55.5% |
|  | Apollo 16 (sample65701, 11) | / | 61.3 |
|  | Apollo 16 (sample67601,30) | / | 29.1% |
|  | Apollo 11 (sample10084) | / | 84.7% |
|  | Apollo 17 (sample70051) | / | 68.4% |
|  | Apollo 17 (sample71501) | / | 73.1% |
| Ref. 3 | Apollo 11 (sample10084) | 90-150 μm | 45.9% |
|  | Apollo 12 (sample12001) | 90-150 μm | 30.9% |
|  | Apollo 12 (sample12030) | 90-150 μm | 54.8% |
|  | Apollo 15 (sample15041) | 90-150 μm | 31.9% |
|  | Apollo 15 (sample15071） | 90-150 μm | 52.6% |
|  | Apollo 17 (sample70181) | 90-150 μm | 14.8% |
|  | Apollo 17 (sample71061) | 90-150 μm | 32.1% |
|  | Apollo 17 (sample71501) | 90-150 μm | 38.4% |
|  | Apollo 17 (sample79221) | 90-150 μm | 51% |
| Ref. 4 | Apollo 16 (sample 61181） | <250μm | 60% |
| Ref. 5 | Apollo 16 (sample 61181) | 90-150μm | 41 % |
| Ref. 2 | Luna 24 (sample 24077) | 90-150μm | 32.2% |
|  | Luna 24 (sample 24109) | 90-150μm | 37% |
|  | Luna 24 (sample 24149) | 90-150μm | 20.4% |
|  | Luna 24 (sample 24174) | 90-150μm | 20.9% |
|  | Luna 24 (sample 24182) | 90-150μm | 13.4% |
|  | Luna 24 (sample 24210) | 90-150μm | 32% |

1. **Water vapor adsorption & desorption of CE5C0400**

Given its small size and strong dipolar character, water may exhibit significant penetrating capabilities within the nano-pores and imperfections of the lunar sample CE5C0400. Therefore, it is crucial to enhance our comprehension of the adsorption properties of water vapor at the nanoscale. Before conducting the water vapor adsorption/desorption isotherm measurements, the lunar sample CE5C0400 was subjected to a degassing process for approximately 100 hours at 423 K, a temperature exceeding the Moon's maximum diurnal temperature. The isotherms were recorded at 303 K using a volumetric BEL BELSORP-max apparatus specifically designed for water vapor adsorption and desorption analysis. Within the pressure range of approximately 0.05< p/p0 < 0.3, the specific surface areas were determined using the Brunauer-Emmet-Teller (BET) method, which assumes a water molecule's surface area to be 0.125 nm^2^.

The representative type II isotherm depicted in Figure S3 indicates water vapor adsorption on the lunar sample, implying a scarcity of pores or a near absence of micropores at the nanometer scale, with the presence of mesopores and macropores facilitating multilayer adsorption. The isotherm at 303 K exhibits a minor and nearly linear incline within the relative pressure range of 0.05 to 0.3. The weak binding suggests there is no chemical reaction between water vapor and the lunar sample at such conditions. The BET analysis of water vapor adsorption on the lunar sample yields an exceedingly low specific surface area of 0.598 m²/g at 303 K. It is important to highlight that the volumetric isotherm measurements are nearing the instrument's sensitivity threshold for such low surface area. The paths of desorption and adsorption display significant hysteresis. The quantity of desorbed water diminishes gradually as it nears the water's saturation vapor pressure, with the hysteresis loop spanning the entire range of relative pressures and failing to close. Consequently, water vapor may desorb at an exceedingly slow rate or may not fully desorb from the lunar sample. These findings may be attributed to the infiltration of water within and around the partial surface layers of the heterogeneous lunar sample. This implies that the desorption of water vapor is significantly impeded by the diffusion of water from the intricate nanoscale cavities of the lunar sample into the external environment.

**Fig. S4** Water adsorption and desorption isotherms of the lunar regolith sample at 303 K.

1. **Numerical simulation**

For the slip flow regime, the slip wall boundary condition was applied at the pore surfaces (equations 5), the pressure boundary condition was set at the bottom inlet, and the outlet pressure was set to 10^-5^ Pa to mimic the high-vacuum environment on the Moon. The continuity equation and N-S equation (equations S1-5) were computed to simulate the volatiles flow through porous agglutinate.

$\rho\left(\mathbf{u}\cdot\nabla\right)\mathbf{u}=\nabla\cdot\left[-p\mathbf{I}+\mathbf{K}\right]$ (S1)

$\nabla\cdot\left(\rho\mathbf{u}\right)=0$ (S2)

$\mathbf{K}=\mu\left(\nabla\mathbf{u}+\left(\nabla\mathbf{u}\right)^{\text{T}}\right)-\frac{2}{3}\mu\left(\nabla\cdot\mathbf{u}\right)\mathbf{I}$ (S3)

$u=A_{1}\lambda\frac{\partial u}{\partial n}$ (S4)

$\lambda=\frac{k_{B}T}{\sqrt{2}\pi d_{kinetic}^{2}P}$ (S5)

where *ρ* is the volatile density, **u** the velocity vector, *μ* the dynamic viscosity, *A*_1_ the reflectivity factor or 1^st^ order slip coefficient and is usually determined by molecular spectroscopy techniques for glass, *λ* the mean free path, $\frac{\partial u}{\partial n}$ the derivative of velocity normal to the boundary, *k*_B_ the Boltzmann constant, *d*_kinetic_ the kinetic diameter of volatile (H_2_O), and *T*, *P* the temperature and mean pressure of the computation domain. For the transition regime, several methods can be applied to simulate the flow in COMSOL including higher order slip flow, diffusion-modified N-S flow, transition flow using the Boltzmann BGK equation, and direct Monte Carlo method. All these methods can yield satisfying results for rarefied gas flow. In this work, the higher order slip flow method was applied to study the volatiles transport in the transition flow regime given its good convergence and proper computation cost. In the simulation, the discretization for the N-S equation of second-order interpolations was applied for velocity, and the first-order slip velocity was set for the wall boundary condition (equations S6):

$u=A_{1}\lambda\frac{\partial u}{\partial n}+A_{2}\lambda^{2}\frac{\partial^{2}u}{\partial n^{2}}$ (S6)

where *A*_2_ is the 2^nd^ order slip coefficient and is usually determined by fitting experimental data, here, *A*_2_ was evaluated by fitting experimental data of N_2_ and corrected by the mean free path of water steam. For the Knudsen diffusion regime, the particle tracing module using the Monte Carlo method was applied to study the transport dynamics of volatile molecules based on Newton’s second law$\frac{d\left(m_{p}\mathbf{v}\right)}{dt}=\mathbf{F}_{\text{t}}$. Initially, the volatile molecules (H_2_O) were confined at the bottom inlet and released under certain temperatures to diffuse through the porous agglutinate. The total number of particles in the system was set to 10^5^ corresponding to the system pressure of $1\times10^{-5}$ Pa. The Molecular mass of the simulated gas is 2.99×10^-26^ kg, and intermolecular force was described by the Lennard-Jones (L-J) potential, where the binding energy J and collision diameter $\sigma=3.3$Å. The gas-wall collision was described by the diffuse scattering model, the velocity distribution function of scattered molecules obeys Maxwell's velocity distribution law concerning the given wall temperature. The equations of motion were evaluated in the simulation with a cutoff length of 1.5 nm and time steps of 10 ps.

In the numerical study, given the extreme conditions of micrometeorites' impact that produce only a small quantity of volatiles, and considering the high escape velocity, it is unlikely for the volatiles to permeate the agglutinate at the temperatures below 1000 K. Meanwhile, to simplify the numerical study, the migration of volatiles is assumed to have a negligible effect on the formed pores of agglutinate. Taking into account that the melting points of basalt and glassy matrix, which are the primary components of agglutinates, exceed 1600 K, and the thermal expansion coefficients vary from $4\times10^{-6}$ to $1.1\times10^{-5}$. Accordingly, for the simulation of slip flow, a pressure range of 8500 Pa to 95000 Pa and a temperature from 1300 K to 1500 K were selected. For the transition flow regime, a pressure range of 85 Pa to 8000 Pa and a temperature from 1300 K to 1500 K were applied with respect to the Kn number from 0.12 to 9.67. In the Knudsen diffusion regime, a total molecule number of $5.44\times10^{5}$ was utilized within the Monte Carlo method to calculate particle motion to balance the computational accuracy and cost. The temperature range of 1000 K to 1500 K were considered to study its influence on the transport of residual volatiles through the agglutinate. A comprehensive summary of the parameters for each flow regime is provided below for reference:

| **Flow Regimes** | **Slip Flow** | **Transition Flow** | **Knudsen Diffusion** |
| --- | --- | --- | --- |
| **Temperature, K** | 1300~1500 | 1300~1500 | 1000~1500 |
| **Pressure, Pa** | 8500~95000 | 85~8000 | 6×10^-4^~9×10^-3^ |
| **Kn** | 0. 01~0.1 | 0.1~10 | 10^5^~10^6^ |

To further understand the diffusion process of residual volatiles at low temperatures after impact, we calculated the transmission probability of volatiles through the agglutinate at temperatures between 300~1300K, as illustrated in Fig. S4. The results show a nonlinear increase of the transmission probability with the environment temperature. And *α* increases approximately 4 times as temperature increases from 300 K to 1300 K at t=1 μs. This further indicates that in the high-temperature, high-pressure environment of impacts, volatiles are unlikely to retain in the porous lunar agglutinate.


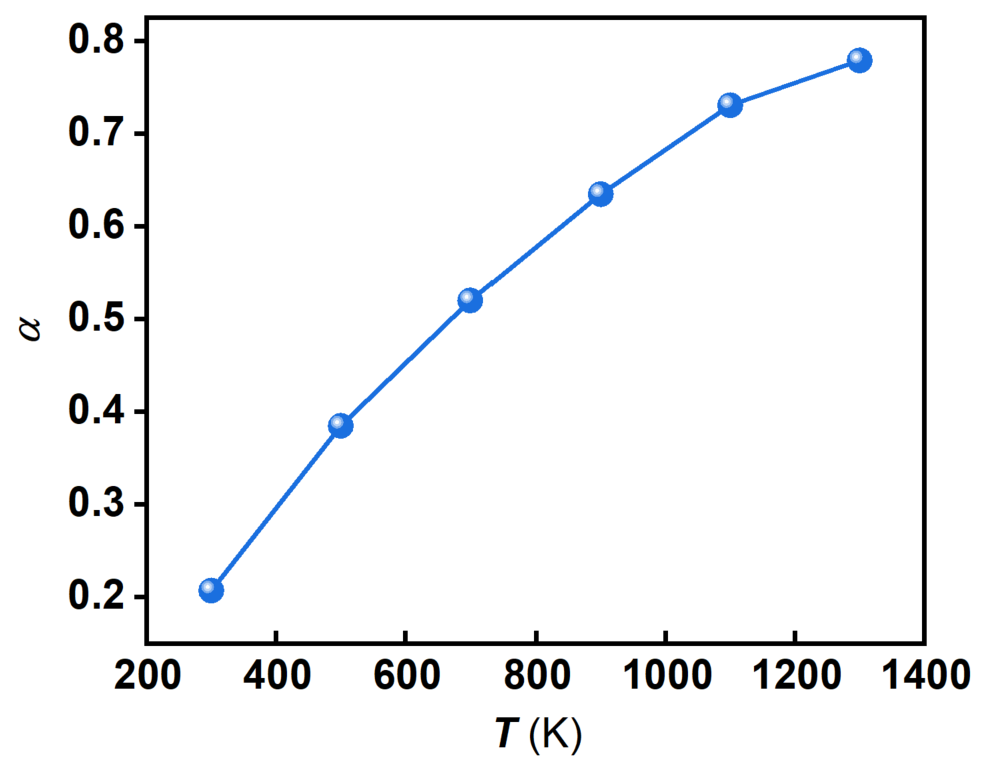


**Fig. S5** Transmission probability with temperature at t=1 μs.

**References**:

1 Desmarais, D. J., Basu, A., Hayes, J. & Meinschein, W. in *In: Lunar Science Conference, 6th, Houston, Tex., March 17-21, 1975, Proceedings. Volume 2.(A78-46668 21-91) New York, Pergamon Press, Inc., 1975, p. 2353-2373.* 2353-2373.

2 Rhodes, J. *et al.* in *In: Lunar Science Conference, 6th, Houston, Tex., March 17-21, 1975, Proceedings. Volume 2.(A78-46668 21-91) New York, Pergamon Press, Inc., 1975, p. 2291-2307.* 2291-2307.

3 Taylor, L. A., Patchen, A., Taylor, D.-H. S., Chambers, J. G. & McKay, D. S. X-ray digital imaging petrography of lunar mare soils: Modal analyses of minerals and glasses. *Icarus* **124**, 500-512 (1996).

4 Morris, R. V. in *In: Lunar and Planetary Science Conference, 9th, Houston, Tex., March 13-17, 1978, Proceedings. Volume 2.(A79-39176 16-91) New York, Pergamon Press, Inc., 1978, p. 2287-2297.* 2287-2297.

5 Houck, K. J. Petrologic variations in Apollo 16 surface soils. *Journal of Geophysical Research: Solid Earth* **87**, A197-A209 (1982).
